# Can Pulsed Electromagnetic Fields Trigger On-Demand Drug Release from High-Tm Magnetoliposomes?

**DOI:** 10.3390/nano8040196

**Published:** 2018-03-27

**Authors:** Martina Nardoni, Elena della Valle, Micaela Liberti, Michela Relucenti, Maria Antonietta Casadei, Patrizia Paolicelli, Francesca Apollonio, Stefania Petralito

**Affiliations:** 1 Department of Drug Chemistry and Technologies, “Sapienza” University of Rome, Piazzale Aldo Moro 5, 00185 Rome, Italy; martina.nardoni@uniroma1.it (M.N.); mariaantonietta.casadei@uniroma1.it (M.A.C.); patrizia.paolicelli@uniroma1.it (P.P.); 2 Department of Information Engineering, Electronics and Telecommunications (DIET), “Sapienza” University of Rome, Via Eudossiana 18, 00184 Rome, Italy; elena.dellavalle@uniroma1.it (E.d.V.); micaela.liberti@uniroma1.it (M.L.); francesca.apollonio@uniroma1.it (F.A.); 3 Department of Anatomical, Histological, Forensic Medicine and Orthopedic Science, “Sapienza” University of Rome, Via A. Borelli 50, 00161 Rome, Italy; michela.relucenti@uniroma1.it

**Keywords:** magneto mechanical trigger, magnetoliposomes, on-demand drug release, magneto nanoparticles, PEMF, non-thermal magnetic field

## Abstract

Recently, magnetic nanoparticles (MNPs) have been used to trigger drug release from magnetoliposomes through a magneto-nanomechanical approach, where the mechanical actuation of the MNPs is used to enhance the membrane permeability. This result can be effectively achieved with low intensity non-thermal alternating magnetic field (AMF), which, however, found rare clinic application. Therefore, a different modality of generating non-thermal magnetic fields has now been investigated. Specifically, the ability of the intermittent signals generated by non-thermal pulsed electromagnetic fields (PEMFS) were used to verify if, once applied to high-transition temperature magnetoliposomes (high-Tm MLs), they could be able to efficiently trigger the release of a hydrophilic model drug. To this end, hydrophilic MNPs were combined with hydrogenated soybean phosphatidylcholine and cholesterol to design high-Tm MLs. The release of a dye was evaluated under the effect of PEMFs for different times. The MNPs motions produced by PEMF could effectively increase the bilayer permeability, without affecting the liposomes integrity and resulted in nearly 20% of release after 3 h exposure. Therefore, the current contribution provides an exciting proof-of-concept for the ability of PEMFS to trigger drug release, considering that PEMFS find already application in therapy due to their anti-inflammatory effects.

## 1. Introduction

Lipid vesicles are considered clinically established systems for the delivery of drugs for nanomedicine applications, due to their biocompatibility and ability to encapsulate both hydrophilic and hydrophobic agents. Using a stimulus to release drugs from carriers at a specific time and location is one of the most sought results of drug delivery research. Typically, such a control is gained by changing the environmental conditions (e.g., ultrasound, UV–vis light, temperature, or pH of the bulk medium) [1,2,3,4,5]. Among these stimuli, electromagnetic fields can offer substantial benefits for nanomedicine and controlled drug delivery as a remote actuation tool [6,7,8]. Most typically, high-frequency alternating magnetic fields (HF-AMF, 50–400 kHz) are used to promote local heating within lipid vesicles encapsulating magnetic nanoparticles (MNPs) either in the membrane or inside the water pool using them as devices for magnetic-controlled delivery of drugs [9,10]. However, the application of these magnetic stimuli may determine damage and secondary effects to the surrounding tissues due to both the temperature increase and to magnetically induced eddy currents, limiting their clinical applicability [11]. Therefore, more recently, the attention has begun shifting to very distinct magnetic field effects exerted on MNPs, namely a magneto-mechanical action, which can be observed with an AMF of much lower intensity and in the absence of heating [12,13,14,15]. Aimed to investigate only the mechanical actuation of magnetic nanoparticles by non-heating AMF, in a previous work, authors demonstrated the possibility to gain a controlled release from high-transition temperature magnetoliposomes (high-Tm MLs) by low-level magnetic stimulation [16,17]. The carrier payload was repetitively released by switching on and off a 20 kHz, 60 A/m magnetic field. The results indicated high reproducibility of cycle-by-cycle release induced by the magnetic-impelled motions driving to the destabilization of the bilayer rather than the phospholipid phase transition or the destruction of the vesicle structure. Therefore, mechanical actuation of magnetic nanoparticles by non-heating magnetic field provides an opportunity to overcome the drawback of heating-magnetic field actuation, creating conditions to consider MNPs and low amplitude magnetic fields prospective powerful therapeutic tools. By the way, AMF are mainly used in laboratory conditions and rarely applied in clinic, nevertheless non-thermal pulsed electromagnetic fields (PEMFs) are already employed in therapy because of their ability to down-regulate specific cytokines in an inflamed environment [18,19,20]. The main goal of this paper is to comprehend if the intermittent signals generated by PEMFs are able to enhance the permeability of high-Tm MLs (Tm = 52 °C) as it occurs with low intensity AMF. The current contribution provides a proof-of-concept for the ability of PEMFs of similar intensity of those used in [16] to trigger on-demand drug release opening a new scenario of synergic dual effect for concrete application in clinics. 

## 2. Materials and Methods 

**Figure 1:**
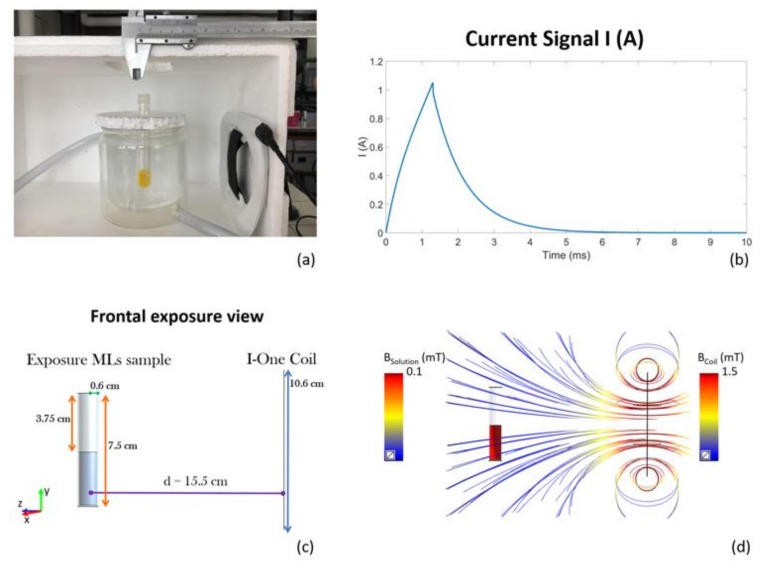
(**a**) pulsed electromagnetic field (PEMF) exposure setup; (**b**) Current signal feeding the coil; (**c**) Model of the high-Tm MLs exposure system in the frontal exposure view; (**d**) Magnetic field distribution in the high-Tm MLs sample given by the magnetic field intensity of the coil (streamlines).

Battery-operated device (I-ONE, IGEA, Carpi, Italy) generating a peak magnetic field of 1.5 mT at the coil, with a repetition frequency of 75 Hz, was tested in our experiments. The device is constituted by a current pulse generator feeding an external coil, which produces the PEMF. The pulsed signal has the following temporal characteristics (see Figure 1b): duration of the active phase of the signal: 1.3 ± 0.1 ms; repetition frequency: 75 Hz (which is equivalent to a repetition time period between two successive pulses of 13.3 ms), amplitude of the current peak value: 1.05 A. 

The high-Tm MLs sample was placed at a distance of 13 cm from the coil (15.5 cm considering the thickness of both the coil and the thermal bath recipient) as shown in Figure 1a, obtaining a magnetic field of 100 µT. Such a distance was chosen with numerical Magneto Quasi-static simulations performed with the Software Sim4Life (V. 3.4, ZMT Zurich MedTech AG, Zurich, Switzerland) at frequency of 250 Hz (the first lobe of the spectral of the signal). The model geometry is reported in Figure 1c. The cuvette has been simulated as a cylinder with 7.5 cm of height and a radius of 0.6 cm, half-filled with a conductive solution (0.049 S/m), which represents the experimentally-measured conductivity of the high-Tm MLs suspension (*N* = 28, std. = 0.005). A fine grid mesh (0.6 mm of resolution) has been adopted for the coil and a more accurate resolution has been chosen for the cuvette discretization (0.4 mm). In Figure 1d the magnetic field streamlines and the B field distribution inside the cuvette are reported, showing a B field of 100 µT inside the solution when 1.5 mT is applied at the coil. The magnetic field appears to be homogeneous inside the sample ensuring a good exposure for the high-Tm MLs suspension. The samples were prepared and characterized as previously reported [16] and here briefly described. We have combined hydrophilic MNPs with the hydrogenated soybean phosphatidylcholine (HSPC) and cholesterol (Chol) to design a suitable carrier model. The obtained liposomes are referred to as high-temperature sensitive magnetovesicles since their membrane can exist only in the ordered state within the experimental temperature interval and neither spontaneous leakage nor thermal responsiveness can occur up to 52 °C. 

Since the liposome membrane does not undergo transition in the experimental temperature conditions, they represent a suitable carrier model to put in evidence the non-thermal effect of the magnetic field on drug delivery. High-Tm MLs were prepared by means of incorporating commercially available carboxymethyl-dextran coated magnetite nanoparticles within the aqueous core of vesicles according the classical film rehydration method followed by extrusion method as reported in [21]. HSPC and Chol (5:1 molar ratio) were dissolved in the minimum volume of chloroform and the organic solution was poured into a round bottom flask. The organic solvent was evaporated under reduced pressure at 60 °C until a thin lipid film was formed on the bottom of the flask. The dry lipid film was then hydrated at 60 °C with 10 mL of HEPES buffer solution (10 mM, Ph = 7.4) containing MNPs and 5-(6) carboxyfluorescein sodium salt (5-(6) CF, 20 mM). Final lipid concentration was 10 mM and magnetite to phospholipid ratio was 0.2 g Fe_3_O_4_/mmol HSPC. Control liposomes (CLs), without MNPs, were prepared by adding 5-(6) CF to the buffer used during the hydration step. The obtained multilamellar vesicles were downsized by sequential extrusion at *T* > Tm to form unilamellar liposomes in a Lipex extruder (Lipex Biomembranes, Vancouver, BC, Canada). This step was performed through polycarbonate membrane filters (Whatman Cyclopore membranes, Whatman International Ltd., Florham Park, NJ, USA) of decreasing pore size (0.8–0.4–0.2 µm) upon ten times to obtain a narrow size distribution. The unencapsulated fluorescent dye and the non-entrapped ferrofluid nanoparticles were removed by size exclusion chromatography with a Sephadex G-50 (Sigma-Aldrich S.r.l., Milan, Italy) column. Hydrodynamic diameter, size distribution and ζ-potential of both CLs and High-Tm MLs were measured with a Zetasizer Nano ZS90 (Malvern Instruments Ltd., Malvern, UK). Phospholipid concentration was determined using the phosphorus colorimetric assay. The magnetite content in magnetoliposomes was determined using the method described by Belikov et al. [22]. All liposome formulations were stored in the dark at 4 °C and used within 1 week. The membrane permeation of the obtained magnetovesicles was evaluated measuring the release behaviour of the self-quenching and membrane impermeable 5-(6) CF, used as a model hydrophilic drug. Specifically, in vitro release of 5-(6) CF from high-Tm MLs and from CLs samples was determined by monitoring marker fluorescence de-quenching through spectrofluorimetry at excitation and emission wavelengths of 492 and 512 nm, respectively. The release was measured both due to an applied PEMF stimulation and under sham-conditions (high-Tm MLs positioned as in Figure 1a but without coil feeding). The release of 5-(6) CF from high-Tm MLs and CLs was evaluated as a function of the PEMF application time, for different time durations: 15, 30, 60 and 180 min. At the end of each series of measurements, all the liposomal vesicles were completely destroyed with addition of a non- ionic detergent (Triton X-100, 30% *w*/*v*) in order to evaluate the residual amount of 5-(6) CF still contained in the samples. A water bath was used to control the environmental temperature (37.0 ± 0.1 °C). Differential scanning calorimetry (DSC) was used to characterize the influence of cholesterol and MNPs on the thermotropic phase behaviour of hydrogenated soybean phosphatidylcholine (HSPC) membrane. The measurements were carried out with a DSC131 (Setaram, Caluire-et-Cuire, France). At least three heating/cooling cycles were performed under nitrogen flow (20 mL/min) by setting an initial isotherm at 30 °C for 300 s, a heating ramp from 30 to 70 °C and a second isotherm at 70 °C for 300 s. The thermograms were recorded at a rate of 5 °C/min. An empty aluminium pan was used as reference. 

## 3. Results

**Figure 2:**
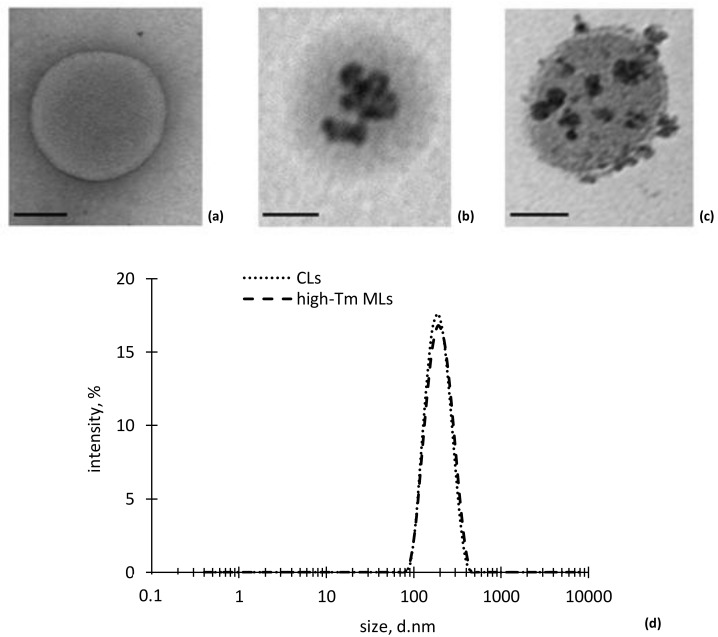
Transmission electron microscope (TEM) images showing hydrogenated soybean phosphatidylcholine (HSPC) liposomes (**a**) conventional and (**b**,**c**) high-Tm MLs. (scale bar: 100 nm); (**d**) DLS measurements of liposome size.

Transmission electron microscopy (TEM) images of CLs and high-Tm MLs structures are shown in Figure 2a–c. CLs and high-Tm MLs exhibited similar size and morphology, which indicates that MNPs interaction, at the lipid/MNPs ratio employed, did not affect vesicles formation and the overall bilayer integrity. TEM images suggest that interaction between MNPs and HSPC lipids leads to magnetoliposomes with hybrid colloidal structure: the iron oxide nanoparticle can either decorate the liposomal surface (Figure 2c) or be internalized inside the vesicles as individual entities or MNPs aggregates (Figure 2b). Free nanoparticles were not observed outside the liposome vesicles throughout the TEM grid, thus indicating the efficacy of the SEC purification step.

No differences were observed in vesicles size between CLs and high-Tm MLs as shown in Figure 2d. Both nanocarriers were arranged in a monomodal distribution with PdI values < 0.200, as reported in Table 1. ζ-potential value of CLs and high-Tm MLs in the buffer medium is, in both cases, negative but the high-Tm MLs show more electronegative character, which suggests the presence of some negatively charged MNPs absorbed onto the external leaflet of the phospholipid bilayer. Hydration step of the dry lipid film during liposome preparation was not influenced by the presence of MNPs in the hydrating medium. In fact, the percentage of lipid molecules found in the self-closing bilayer of high-Tm MLs only partially decreased respect to control liposomes (>80%). Finally, to probe the membrane permeation and release behaviour of high-Tm MLs, 5-(6) CF was used as a model hydrophilic drug, with satisfactory values of 5-(6) CF loading efficiency expressed as captured volume of marker bulk solution (20 mM) to lipid ratio (µL/mg structured HSPC), which was not limited by the co-loading of MNPs inside vesicles (Table 1). After SEC purification, the lipid and the magnetite content were determined and the encapsulation efficiency was calculated as g of iron oxide per mmol of lipid and the results reported in Table 1. 

**Figure 3:**
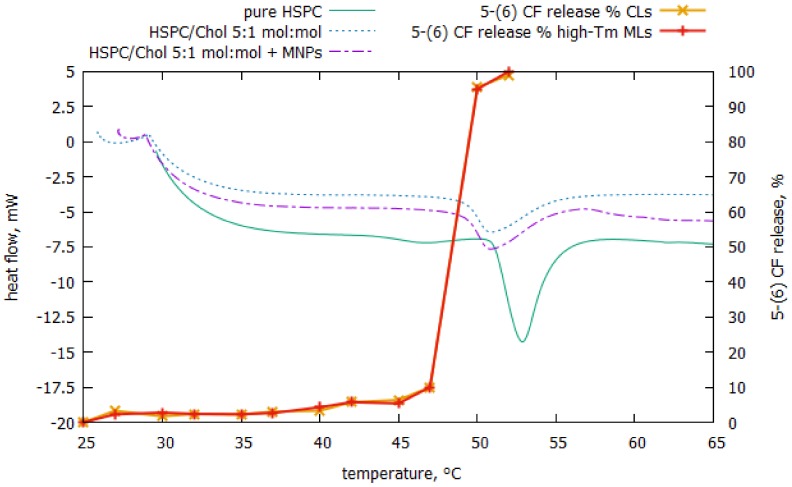
5-(6) CF cumulative release from high-Tm MLs (red points-lines) and CLs (orange points-line) as a function of temperature from 25 to 52 °C and DSC scanning profile of the melting process of HSPC/Chol 5:1 mol:mol mixture with or without MNP_S_.

In the thermogram reported in Figure 3, pure HSPC membranes exhibit two endothermic peaks at 46.6 °C and 52.8 °C corresponding to the pre- and main transition temperature (Tm), respectively. 

When cholesterol (HSPC/Chol 5:1 mol:mol) was added to HSPC, the endothermic peak corresponding to the main transition of the phospholipid became smaller and broader and the peak corresponding to the pre-transition disappeared. The position of the main transition peak of the mixture shifted toward lower temperatures, anyway liposomes with cholesterol can be still considered as high-Tm membrane vesicles since within our experimental conditions they can exist only in the ordered state. Neither spontaneous leakage nor thermal effects can occur up to 50 °C. Our results are in agreement with those reported by Kitayama and co-workers in [23]. 

We have also investigated the effect of the MNPs on the phase behaviour of HSPC/Chol 5:1 mol:mol mixture. As evident in the Figure 3 MNPs did not hinder the lipid ordering and phase behaviour of the investigated bilayer. Moreover, the MNPs did not influence the thermal leakage behaviour of high-Tm MLs compared to CL. 5-(6) CF leakage from high-Tm MLs and CLs was examined as a function of temperature from 25 to 52 °C. As evident from the results reported, both the high-Tm MLs and CLs exhibited maximum changes in 5-(6) CF leakage at temperatures corresponding to the melting transition. These results confirm the agreement between the leakage and phase transition peaks.

The release of 5-(6) CF from high-Tm MLs was evaluated as a function of the PEMF application time.

**Figure 4:**
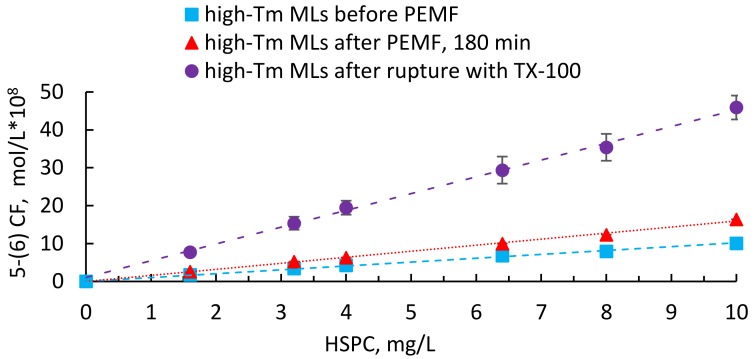
Release data of 5-(6) CF from high-Tm MLs before and after PEMF exposure. The results are reported as molar concentration of marker per different lipid concentrations and compared with the total amount of fluorescent dye released after complete destruction of high-Tm MLs with Triton X-100. The calibration curve, ${\;F}_{A.U}=1.47\times 10^{9}\left[5-\left(6\right)CF\frac{mol}{L}\;\right]+4.18$ ($R^{2}=1.00$), was used to establish a relationship between 5-(6) CF fluorescent intensity and dye concentration (as reported in S.I.).

Figure 4 displays the release behaviour of high-Tm MLs reported as concentration of 5-(6) CF released per different lipid concentrations before and after 180 minutes of continuous PEMF exposure. At the end of each series of measurements, all the liposomal vesicles were completely destroyed with addition of a non- ionic detergent in order to evaluate the residual amount of 5-(6) CF. According to the results, PEMFs stimulation modify the membrane permeability, as indicated by the marker leakage, which reaches about 17% after 180 min. 

**Figure 5:**
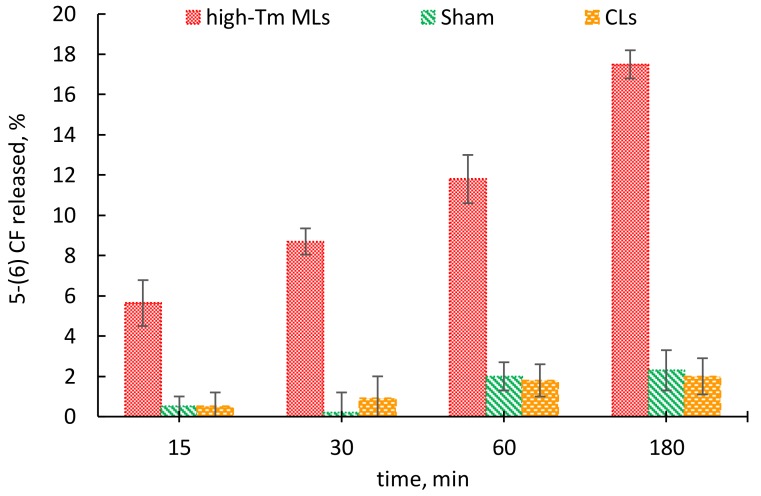
Percentage of the 5-(6) CF release from high-Tm MLs, CLs and Sham at 15, 30, 60 and 180 min.

The increase of the release as a function of exposure duration is an indicator of a dose-response effect as reported in Figure 5, where moving from 15 to 180 min of exposure the fluorescent release presents an overall three-fold increase. 

Nevertheless, it is important to say that the application of PEMF did not lead to the liposomes rupture.

## 4. Discussion

Over the last years, magnetoliposomes have been extensively investigated for controlled drug release. The localized heating produced by movements of MNPs under a magnetic field of sufficient intensity and frequency act as the main trigger for drug release. In fact, when magnetic nanoparticles are combined with thermosensitive lipids, the release of the drug from the hybrid thermosensitive liposome/nanoparticle assemblies can be essentially attributed to the caloric effect resulting in the phase transition of the thermosensitive-liposome. In particular, the magnetically induced heat is transferred to the entire carrier causing temperature increases from physiological temperature to tolerable hyperthermia (41–46 °C) with structural changes in the lipid bilayer that is designed to shift from a gel to liquid phase when this increase in temperature occurs. While the magnetically induced hyperthermia is the main applied approach in controlled drug delivery, it may indiscriminately affect every surrounding tissue, because it is generally very tricky to keep temperature inside the therapeutic window. 

Therefore, the magneto-mechanical approach based on the vibration or rotation of iron oxide particles not associated with heat generation is of more wide application. In fact, magnetic field trigger at frequencies and strength that are several orders of magnitude lower than those needed for the magnetic thermal approach, gives some supplementary advantages. In particular, low amplitude (<100 A/m, 20 kHz) AMF are able to produce mechanical forces from magnetic nanoparticle, which generate transient deformations in the nanometre scale within the liposomal membrane [16]. Similarly, PEMFs proved to be an effective remote trigger. In fact, the obtained results demonstrate that high-Tm MLs respond to PEMF at temperature well below the main transition temperature of the bilayer (Tm = 52 °C). Also with this modality of magnetic field generation, the payload release from high-Tm MLs was due to the magnetic-impelled motions of the MNPs, which drive the destabilization of the bilayer without destruction of the liposome structure or modification of the arrangements of the phospholipids. Furthermore, the high-Tm MLs were stable and able to preserve their physical properties over time, for at least 30 days.

Overall, these achievements may be used to design novel PEMF-sensitive drug delivery systems taking advantage of the mechanical stress induced on the liposome membrane. In particular, the combination of magneto-carriers and PEMFs is an exciting dual approach, where synergistic properties of both modalities could be effectively utilized for inflammatory reduction and remotely-activated drug delivery. In particular, PEMFs has been demonstrated to exert an anti-inflammatory effect resulted in early pain control and enhanced functional recovery in the knee diseases, reducing knee osteoarthritic lesion progression with long term positive benefits for patients [24,25]. At the same time, it is well known that a single-dose intra-articular administration of liposomes within a synovial joint, can be used to treat the inflammatory process [26,27]. Therefore, a novel concept of enhanced localized treatments could be proposed combining the functionalities of the magneto-mechanical actuation and the healing properties of a non–thermal magnetic field. Both approaches could work in close synergy with drug-loaded nanocarriers, able to control drug release by application of a remote magnetic field. In this way, the efficiency of anti-inflammatory therapies can be maximized in future medical applications.

## Figures and Tables

**Table 1:** Physical characterization of liposomes: the hydrodynamic diameter (Z-average), PdI values and ζ-potential values for control liposomes (CLs) and magnetic (high-Tm MLs) liposomal preparations were determined immediately after size exclusion chromatography (SEC) purification. Loading efficiency of 5-(6) CF and iron oxide are also reported.

Sample	CLs	High-Tm MLs
Hydrodynamic diameter (nm)	220.9 ± 22.4	240.9 ± 26.6
PdI	0.046 ± 0.028	0.131 ± 0.031
ζ-potential (mV)	−10.28 ± 1.43	−15.42 ± 1.51
5-(6) CF loading efficiency (µL/mg HSPC)	2.29 ± 0.26	1.84 ± 0.13
Fe_3_O_4_ loading efficiency (g/mmol HSPC)	-	0.2

## Supplementary Materials

Supplementary File 1
