# Supplementary material for: Can Pulsed Electromagnetic Fields Trigger On-Demand Drug Release from High-Tm Magnetoliposomes?
Source: Nanomaterials (Basel). 2018 Mar 27;8(4):196. doi: 10.3390/nano8040196 (PMC5923526; doi:10.3390/nano8040196)
Supplement: Supplementary File 1 [file nanomaterials-08-00196-s001.pdf]

nanomaterials

Supplementary information

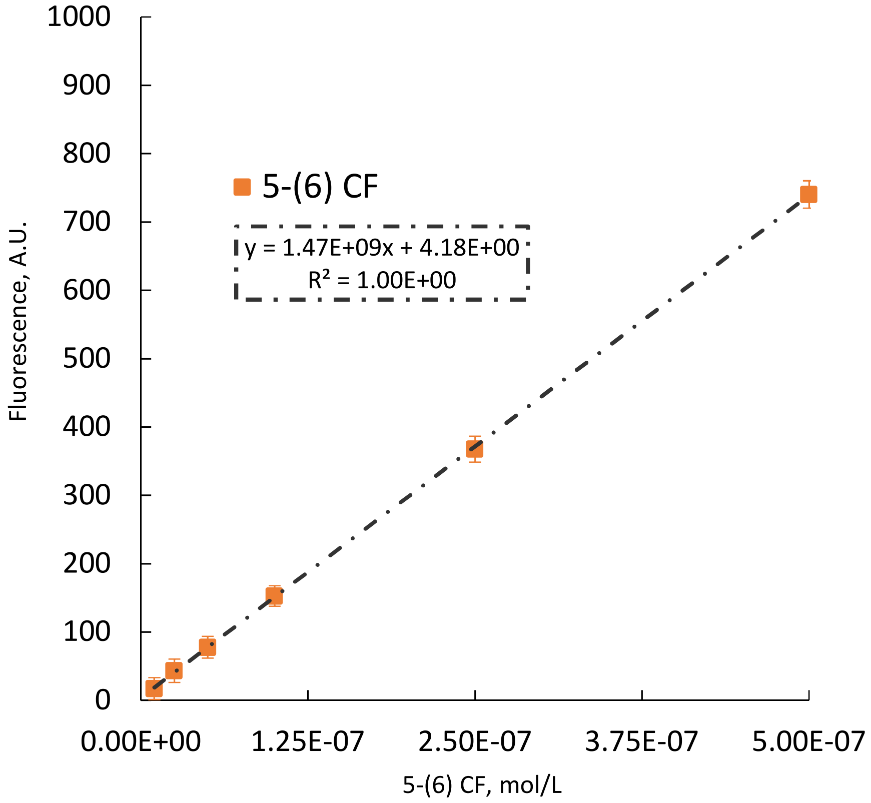

Figure SI. Calibration curve of 5-(6) CF with the linear regression mentioned in Figure 4.
